# Development and validation of a droplet digital PCR assay for sensitive detection and quantification of *Phytophthora nicotianae*

**DOI:** 10.3389/fpls.2025.1573949

**Published:** 2025-04-25

**Authors:** Yuanyuan Liu, Jiali Li, Zining Guo, Chao Feng, Yunhua Gao, Danmei Liu, Di Wang

**Affiliations:** ^1^ School of Life Sciences, Shanxi University, Taiyuan, China; ^2^ Center for Advanced Measurement Science, National Institute of Metrology, Beijing, China; ^3^ School of Pharmacy and Bioengineering, Chongqing University of Technology, Chongqing, China; ^4^ College of Bioengineering, Tianjin University of Science and Technology, Tianjin, China; ^5^ Key Laboratory of Tobacco Pest Monitoring Controlling & Integrated Management, Tobacco Research Institute of Chinese Academy of Agricultural Sciences, Qingdao, China

**Keywords:** *Phytophthora nicotianae*, droplet digital PCR, quantitative PCR, pathogen detection, plant disease management

## Abstract

Tobacco black shank (TBS) disease, caused by *Phytophthora nicotianae* (*P. nicotianae*), poses a significant threat to global agriculture and results in substantial economic losses. Traditional methods, like culture-based techniques and quantitative polymerase chain reaction (qPCR), aid pathogen identification but can be less sensitive for complex samples with low pathogen loads. Here, we developed and validated a droplet digital PCR (ddPCR) assay with high sensitivity and specificity for detecting *P. nicotianae*. ddPCR and qPCR revealed comparable analytical performance including limit of blank (LoB), limit of detection (LoD), and limit of quantitation (LoQ). For the 68 infectious tobacco root samples and 145 surrounding soil samples, ddPCR demonstrated greater sensitivity, with a higher positive rate of 96.4% vs 83.9%. Receiver operating characteristic (ROC) analysis showed an area under the curve (AUC) of ddPCR was 0.913, compared to 0.885 for qPCR. Moreover, ddPCR provided better quantification accuracy for low pathogen concentrations in soil, suggesting better tolerance to potential PCR inhibitors in soil. These results highlight ddPCR as a robust and reliable tool for early diagnosis in complex samples, offering a valuable tool for improving disease management strategies.

## Graphical Abstract

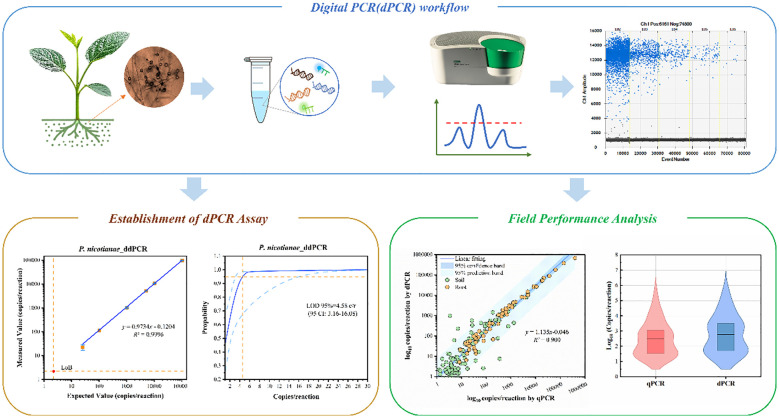

## Introduction

*Phytophthora nicotianae* (*P. nicotianae*) is a highly destructive soil-borne oomycete pathogen responsible for significant yield losses and economic damage in crops such as peppers, strawberries, and tobacco worldwide (Li et al., 2013; Vanegas-Villa et al., 2020; Burgess et al., 2021). The pathogen causes symptoms such as wilting, root rot, and stem lesions, which can lead to plant death if not managed effectively (Raftoyannis and Dick, 2006; Baysal-Gurel et al., 2022; Pandey, 2023). Successful plant disease management relies on an integrated approach combining multiple strategies (Zhang et al., 2018; Ma et al., 2024). Accurate pathogen detection is fundamental to implementing appropriate management measures, such as crop rotation, resistant varieties, and fungicide applications, thereby improving enhancing disease control efficiency and sustainability.

Current molecular diagnostic methods, such as conventional quantitative polymerase chain reaction (qPCR), are widely used in pathogen detection. Li et al (Li et al., 2011; Blaya et al., 2015). established a qPCR method for the ITS region of *P. nicotianae* and achieved efficient detection. In addition, several emerging molecular technologies, including metagenomic sequencing and digital PCR, serve as valuable complementary diagnostic tools. By examining entire microbial genomes or metagenomes, metagenomics has become an important approach for exploring pathogen diversity, tracking emerging strains, and investigating host-pathogen dynamics. However, the routine diagnostic and large-scale monitoring applications of this method remain limited due to its complex protocols, dependence on advanced bioinformatics tools, and high costs (Kalantar et al., 2020; Piombo et al., 2021; Roman-Reyna and Crandall, 2024).

Digital PCR (dPCR) has emerged as a valuable method for molecular diagnostics, offering an optimal balance between sensitivity and accuracy (Rotondo, 2020; Hou et al., 2023). It enables absolute quantification of nucleic acids based on Poisson distribution by analyzing the presence or absence of fluorescent signals in thousands of microreaction units (Hu et al., 2021; Meng et al., 2022). Due to the end-point detection and excessive dilution, only minimal optimization for dPCR assay is necessary, whereas qPCR requires the relatively complex adjustments to obtain high amplification efficiency to ensure sensitivity and accuracy (Zhao et al., 2021; Damgaard and Treebak, 2022). Therefore, dPCR technology has been applied in a variety of applications, including quantification of cancer markers (Development of DDPCR blood-based diagnostic tests that simultaneously measure mRNA expression from immune and cancer cells; Riudavets et al., 2021), determination of viral load (Rotondo, 2020; Deng et al., 2024), and environmental monitoring (Molecular ecology resources; Zhu et al., 2020). In addition, it is relatively-tolerant to PCR inhibitors in complex environmental matrices such as soil and plant tissues (Dingle et al., 2013; Maheshwari et al., 2017; Zafeiriadou, 2024). These characteristics imply it is a promising tool for pathogen detection and quantification in plant pathology.

In this study, we developed a droplet digital PCR (ddPCR) method for detecting *P. nicotianae* and evaluated its analytical performance. We further compared ddPCR and qPCR using 213 field-collected samples, analyzed diagnostic performance through ROC curves to validate sensitivity and diagnostic accuracy. Additionally, pathogen loads were quantified and compared between the two methods.

## Materials and methods

### Sample collection and DNA extraction

Tobacco samples, including root tissue and rhizosphere soil, were collected from tobacco fields in Qingdao, Shandong Province, China. Approximately 50 mg of root tissue was ground in liquid nitrogen, and total DNA was extracted using the DNeasy Plant Mini Kit (Qiagen, Germany) following the manufacturer’s protocol. For soil samples, around 5 grams of rhizosphere soil—defined as soil in close contact with the roots—was collected, and DNA was extracted using the DNeasy PowerSoil Kit (Qiagen, Germany) in accordance with the manufacturer’s instructions. The quality and concentration of the extracted DNA from all samples were assessed using a NanoDrop spectrophotometer (Thermo Fisher Scientific, USA).

### ddPCR and qPCR methods

The primer/probe used in the study designed by Li at al. Nic-Forward: 5’-CCTATCAAAAAACAAGGCGAACG-3’, Nic-Reverse: 5’-CAGAGACTTTCGTCCCCACAGT-3’, and Nic-Probe: 5’-CTTCGGCCTGATTTAGTAGT-3’. For the ddPCR assay, experiments were conducted using the QX200 Droplet Digital PCR System (Bio-Rad, USA). The 20 µL reaction mixture consisted of 10 µL of 2× ddPCR Supermix for Probes (Bio-Rad), 1 µL of each primer (final concentration 500 nM), 0.5 µL of the probe (final concentration 250 nM), 2 µL of template DNA, and nuclease-free water to achieve the final volume. The probe was labeled with FAM (6-carboxy-fluorescein) at the 5’ end and a black hole quencher 1 (BHQ1) at the 3’ end (Sangon Biotech). Droplets were generated using the QX200 Droplet Generator (Bio-Rad) following the manufacturer’s protocol. The droplets were then transferred to a 96-well PCR plate, sealed with pierceable foil, and thermally cycled in a Veriti™ 96-well thermal cycler (Applied Biosystems, USA). The cycling protocol included an initial denaturation at 95°C for 10 minutes, followed by 45 cycles of denaturation at 94°C for 30 seconds and annealing/extension at 58°C for 1 minute, with a final extension at 98°C for 10 minutes and a hold at 4°C. After amplification, the droplets were read using the QX200 Droplet Reader (Bio-Rad), and data were analyzed with QuantaSoft software (Bio-Rad).

For qPCR, reactions were performed using the Light Cycler 480 II (Roche Applied Science, Germany). Each 20 µL reaction mixture contained 10 µL of 2× Probe qPCR MasterMix (TianGen, China), 1 µL of each primer (final concentration 500 nM), 0.5 µL of the probe (final concentration 250 nM), 2 µL of template DNA, and nuclease-free water to complete the final volume. The thermal cycling conditions for qPCR were identical to those used for ddPCR, and references to Li et al.

### Specificity of ddPCR assay

The specificity of the primers and probes was assessed using a panel of 15 closely related *Phytophthora* spp. and other pathogens, including *P. capsici*, *P. cactorum*, *P. ramorum*, *P. infestans*, *P. citrophthora*, *P. cryptogea* A1, *P. cryptogea* A2, *P. cinnamomi*, *Pythium* spp., *P. ultimum*, *P. aphanidermatum*, *Pythium myriotylum*, *Verticillium albo-atrum*, *Verticillium dahliae*, *Pythium helicoid.*

### Analytical performance assessment

The dynamic range was determined by a linear fit using a series of dilution samples, with nine replicates per concentration. To establish the limit of blank (LoB), 60 measurements were performed on three blank samples at different times. Blank measurements were defined as reactions containing nucleic acid-free water instead of a DNA template, conducted under the same ddPCR conditions as the test samples (Dong et al., 2020).

The limit of quantification (LoQ) was determined by conducting 20 measurements across five serial dilutions of samples. LoQ is defined as the lowest target DNA concentration that can be reliably quantified by the ddPCR assay with acceptable precision, indicated by a coefficient of variation (CV) of less than 25% (Kralik and Ricchi, 2017; Dong et al., 2020).

The limit of detection (LoD) represents the lowest concentration at which the ddPCR assay can detect target DNA with 95% confidence interval (CI). LoD was determined by performing 70 measurements across seven low-concentration dilution series and analyzing the data using probit regression, following the EP17-A guidelines (Tholen et al., 2004).

### Statistical analysis

All statistical analyses were performed using OriginPro 2024 (version 10.1.0.178). Data were reported as mean ± standard deviation (SD), unless otherwise specified. The Shapiro-Wilk test was used to assess the normality of data distribution. Correlation analysis was conducted using either Pearson or Spearman methods, based on the data distribution. ROC curves were generated to evaluate the diagnostic performance of qPCR and ddPCR platforms, and the AUC values were calculated. A p-value of < 0.05 was considered statistically significant.

## Results and discussions

### Optimization of ddPCR method

**Figure 1:**
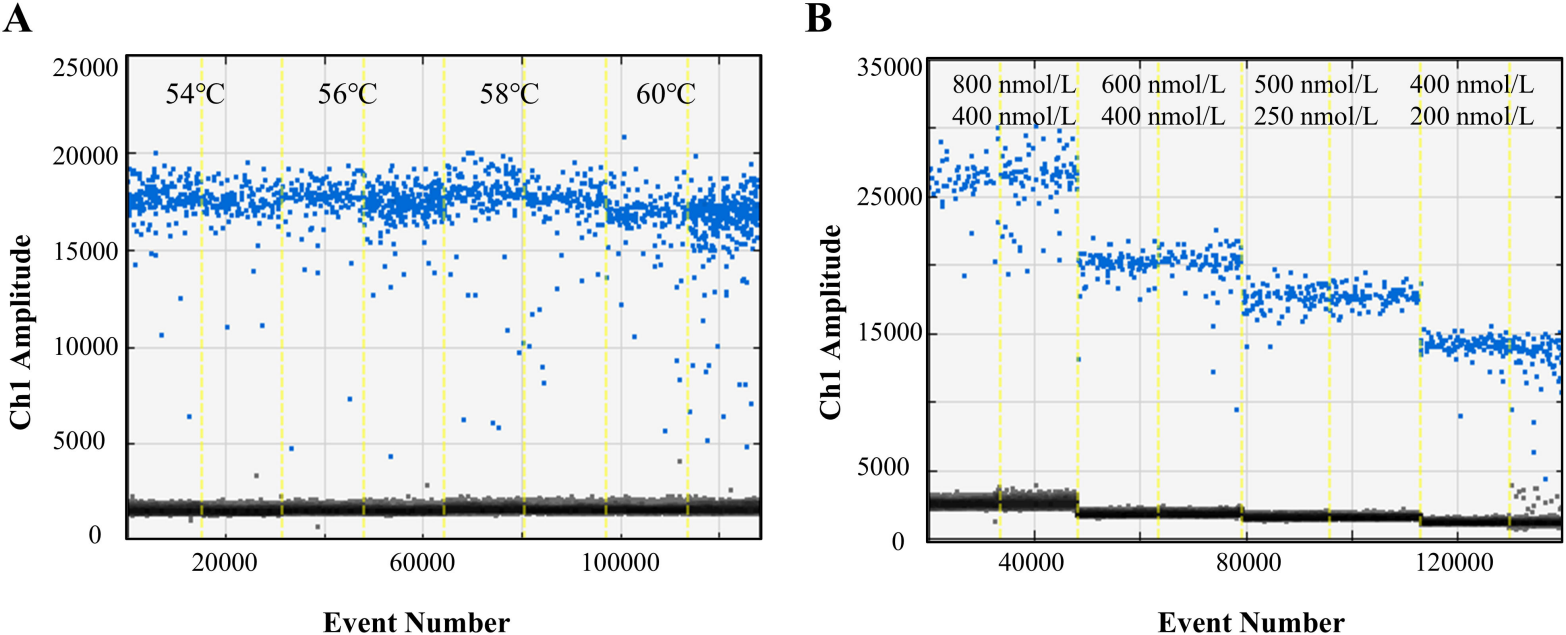
Optimization of ddPCR parameters. **(A)** Optimization of ddPCR reaction conditions from 54°C to 60°C. **(B)** Optimization of primer/probe concentration in ddPCR reaction system. The Blue and Gray droplets represent positive and negative droplets respectively.

The ddPCR reaction conditions and system were optimized to achieve optimal performance. The assay performed well across a range of annealing temperatures (**Figure 1A**). An annealing temperature of 58°C was selected as it provided clear separation of positive and negative droplets, with minimal “rain” (droplets with intermediate fluorescence values). Additionally, the reaction system was also optimized, and the primer and probe concentrations were determined to be 500 nM and 250 nM, respectively (**Figure 1B**).

### Analytical specificity

The specificity of the ddPCR assay was confirmed by testing in a panel of closely related *Phytophthora* species and other pathogens. No cross-reactivity was observed, demonstrating that the primers and probes specifically amplified *P. nicotianae* DNA without interference from other pathogens (**Supplementary Figure S1**).

### Dynamic range and limit of blank

**Figure 2:**
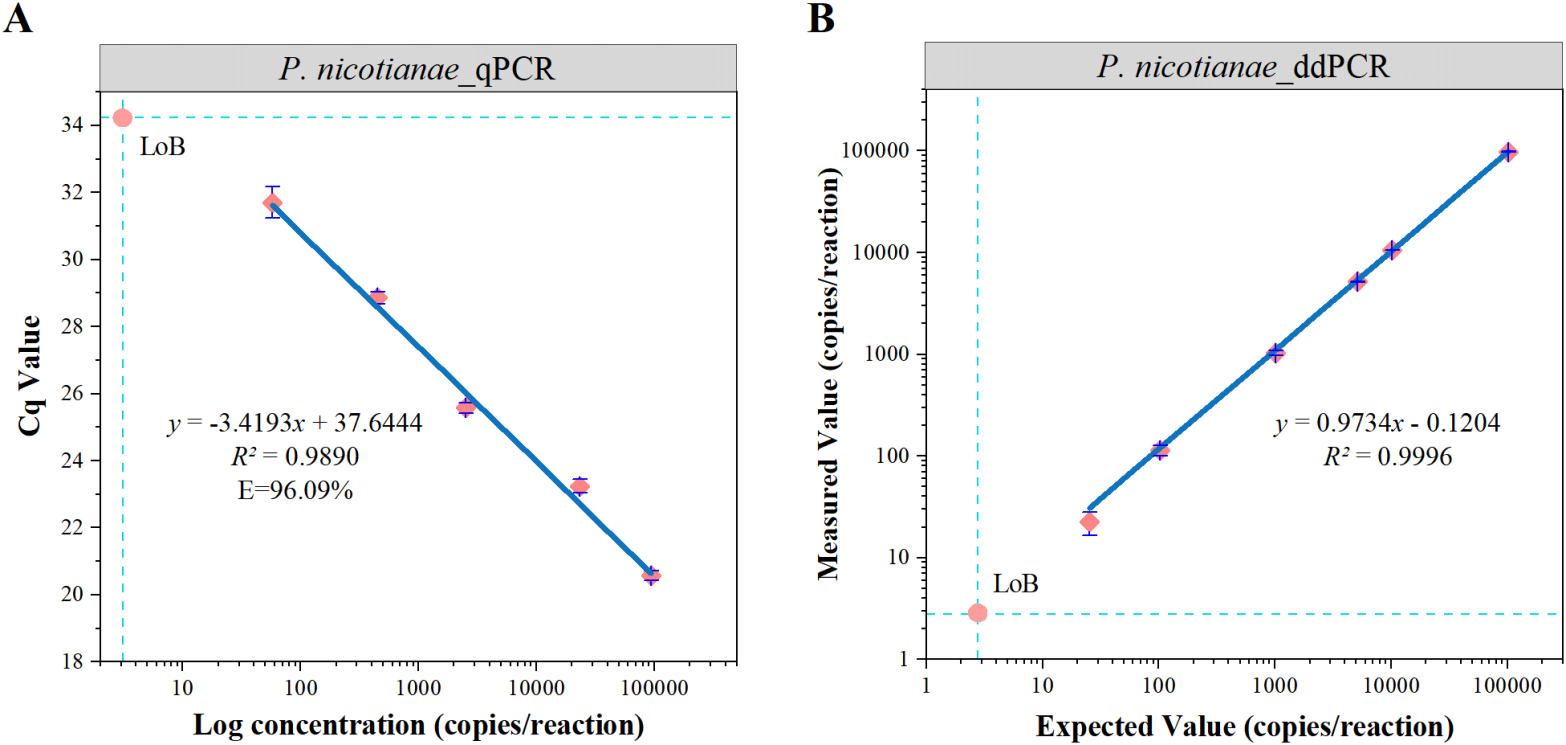
Dynamic range and LoB of qPCR and ddPCR analyses **(A, B)**. The blue line indicates linear regression of the detected value against the expected value. The error line indicates the standard deviation and the intersection of the dashed lines is the LoB.

The dynamic range of the ddPCR assay was determined by serially diluting standard genomic DNA samples of *P. nicotianae* (10¹ to 10^5^ copies/reaction) using the gravimetric method. The assay exhibited good linearity across five orders of magnitude, with R² values of 0.989 for qPCR and 0.999 for ddPCR (**Figures 2A, B**).

The LoB was determined based on 60 measurements from six blank samples. Although the distribution of the 60 blank controls was expected to conform to a normal distribution, the Shapiro-Wilk test revealed that neither qPCR (W = 0.375, P < 0.05) nor ddPCR (W = 0.408, P < 0.05) followed a normal distribution (**Supplementary Figure S2**). Consequently, a non-parametric approach was adopted following the EP17-A guidelines (EP17-A protocols for determination of limits of detection and limits of quantitation; approved guideline), and the LoB was calculated based on the 95th percentile of the measurements: LoB = X57 + 0.5 × (X58 − X57). **Supplementary Table S2** presents the 15 highest blank values for each target. Linear interpolation between the 57th and 58th values yielded an estimated LoB of 2.8 copies/reaction for ddPCR and 3.1 copies/reaction for qPCR (**Figures 2A, B**).

### Limit of Quantification and Detection

To evaluate LoQ, standard gDNA *P. nicotianae* samples were series diluted from 50 to 3 copies/reaction. Both methods showed good accuracy, but ddPCR showed less bias between expected and measured values across all the tested samples (**Table 1**). While the LoQ for both ddPCR and qPCR analysis was 8 copies/reaction, ddPCR showed lower bias (0.9%) and RSD (10.4%), indicating higher accuracy. At 3 copies/reaction (below the LoQ), the RSD of ddPCR (29.3%) was significantly lower than that of qPCR (49.6%).

**Table 1:** LoQ of ddPCR and qPCR.

	Expected copies/reaction	Measured copies/reaction				Mean	Bias (%)	SD	RSD (%)
		Run 1	Run 2	Run 3	Run 4				
ddPCR	50.0	57.0	53.0	48.0	48.0	51.5	3.0	3.8	7.3
	25.0	28.5	26.5	24.0	24.0	25.8	3.0	1.9	7.3
	12.5	14.7	12.7	11.6	8.8	12.0	-4.4	2.1	17.8
	8.0	7.7	7.9	6.9	9.2	7.9	-0.9	0.8	10.4
	3.0	3.2	4.5	2.3	2.3	3.1	2.5	0.9	29.3
qPCR	50.0	44.1	48.2	45.1	52.0	47.3	-5.3	3.1	6.5
	25.0	18.2	18.9	21.4	19.9	19.6	-21.6	1.2	6.3
	12.5	14.7	13.0	15.4	13.6	14.2	13.3	0.9	6.5
	8.0	6.9	9.6	10.5	12.6	9.9	23.6	2.1	20.9
	3.0	4.2	1.3	2.7	2.0	2.6	-14.5	1.1	41.6

**Figure 3:**
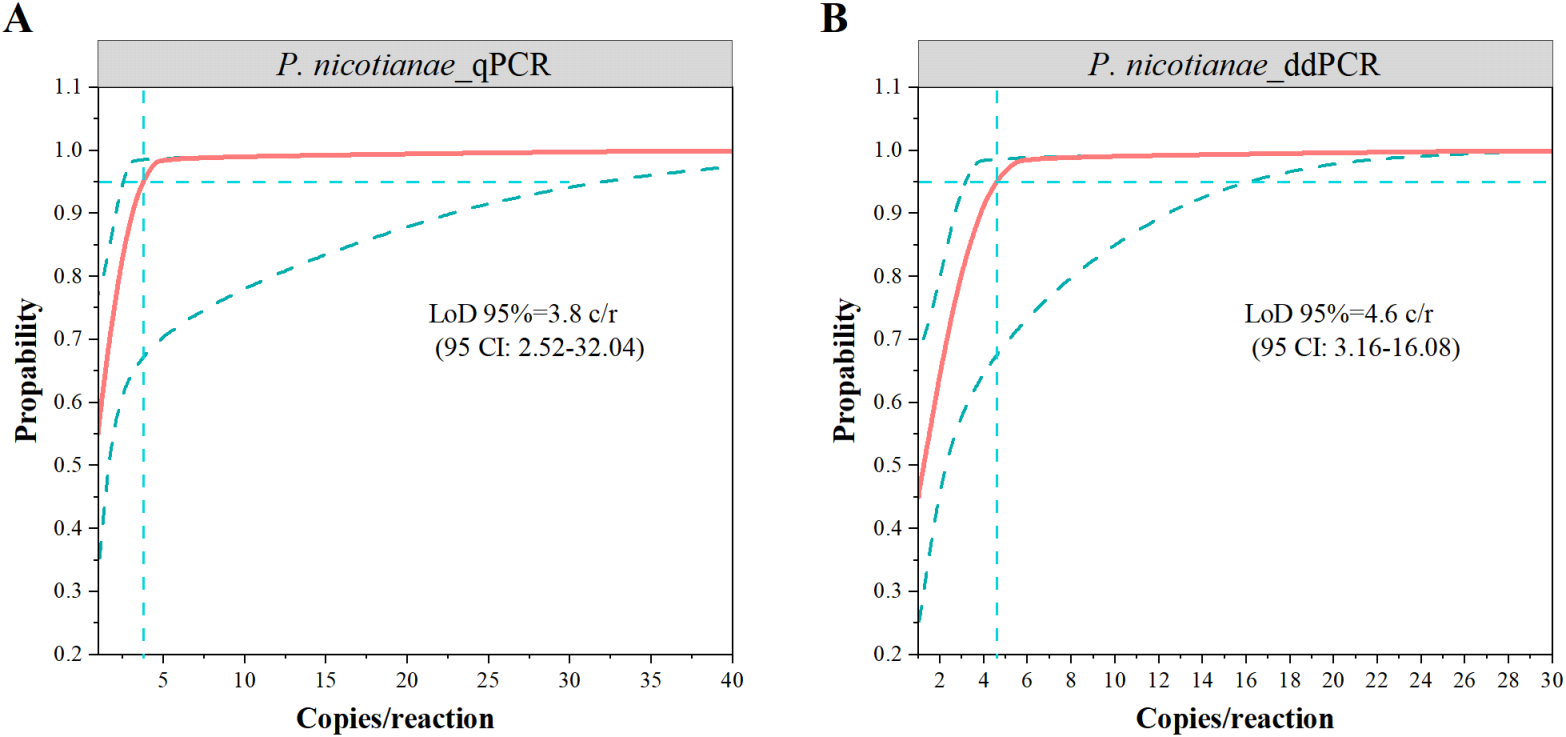
The limits of detection of 95% (LoD95%) via probit analysis for qPCR **(A)** and ddPCR **(B)**. The vertical dotted lines indicated LOD and values are denoted with 95% confidence intervals in parenthesis.

Seventy measurements from seven low-concentration samples (1 to 50 copies/reaction) were analyzed by probit analysis to determine the LoD (**Figures 3A, B**). The results showed a LoD95% of 4.6 (95% CI: 3.2 ~ 16.1) copies/reaction for ddPCR and 3.8 (95% CI: 2.5 ~ 32) copies/reaction for qPCR, indicating similar detection sensitivity between the two methods. Notably, the upper limit of the 95% CI for ddPCR was lower, suggesting higher positivity rates for low-concentration samples.

### Diagnostic performance of qPCR and ddPCR

**Figure 4:**
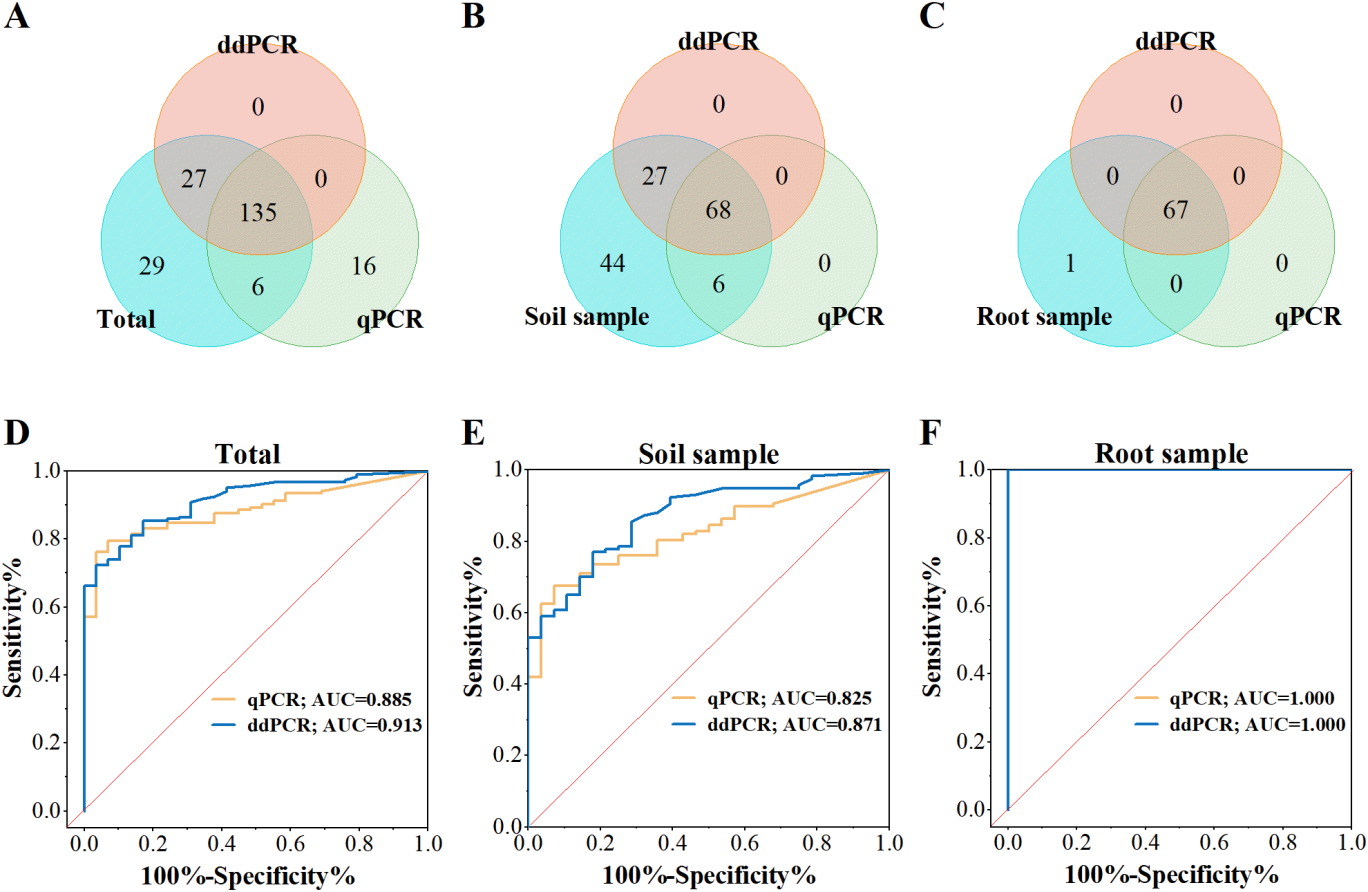
Diagnostic performance of ddPCR and qPCR for *P. nicotianae*. **(A-C)** Venn diagrams comparing ddPCR and qPCR results for field-collected samples. **(D-F)** ROC analysis of ddPCR and qPCR. The red diagonal line represents the random prediction baseline. (Each sample was tested in duplicate.).

To assess diagnostic performance in crops, a total of 213 field samples were analyzed, including soil and root samples. The infection status of these samples was previously verified for the presence or absence of *P. nicotianae* through the colony culture method. The cutoff values for distinguishing positive and negative samples were determined as 3.1 copies/reaction for ddPCR and 5.9 copies/reaction for qPCR, calculated using the maximum Youden index. ddPCR demonstrated significantly higher detection sensitivity compared to qPCR for these samples (**Figures 4A, C**). Among the 101 positive soil samples, 68 (67.3%) were identified as positive by both methods, while ddPCR detected an additional 27 samples (ranging from 3.4 to 486.4 copies/reaction, mean: 63.9 copies/reaction) that were missed by qPCR (**Figure 4B**). For the 68 root samples, 67 were consistently identified as positive by both methods (**Figure 4C**).

ROC curve analysis further evaluated the diagnostic performance of both methods (**Figures 4D-F**). The AUC value for ddPCR (0.913) was higher than that of qPCR (0.885) (**Figure 4D**). For soil sample, ddPCR achieved an AUC of 0.871, outperforming qPCR (0.825) (**Figure 4E**). In contrast, both methods showed perfect accuracy (AUC = 1.000) for root samples (**Figure 4F**). The overall performance difference was largely attributed to soil samples.

### Quantification of *P. nicotianae* in field-collected samples

**Figure 5:**
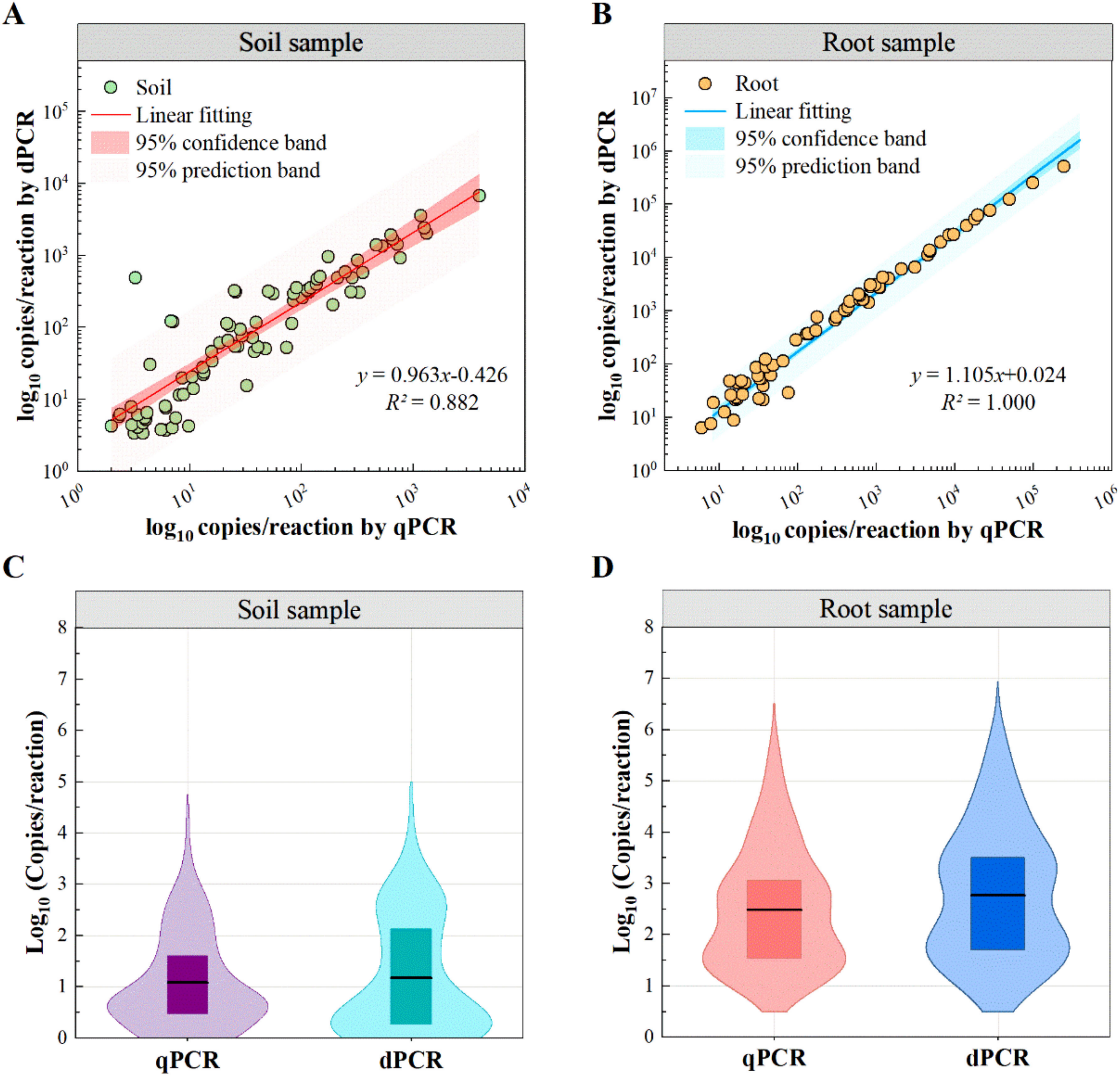
Distribution of quantified *P. nicotianae*. by qPCR and ddPCR. **(A, B)** Correlation between qPCR and ddPCR results. **(C, D)** Violin plots showing the distribution of target copy numbers detected by qPCR (purple/red) and ddPCR (cyan/blue) in the samples. Boxes indicate the interquartile range (25%-75%) and the black horizontal line indicates the mean.

The quantitative results of 68 positive soil samples and 67 positive root samples display a high correlation coefficient between qPCR and ddPCR (Pearson r>0.80; **Figures 5A, B**). However, the correlation coefficient for soil samples (R^2^ = 0.882) was lower compared to that of root samples (R^2^ = 1.000).In addition, the two methods’ quantitative results showed a greater bias for low-concentration samples, as reported in other studies (Sidstedt et al., 2020; Wang et al., 2022).Thus, we consider that qPCR is an effective technology, while its reliability could decrease when testing the complex low-quantity samples compared with digital PCR.

To further explore this difference, violin plot distributions demonstrate a high consistency of patterns between the two methods in soil and root samples (**Figures 5C, D**). However, measured values of ddPCR were significantly higher than qPCR (mean: 7176.4 vs 2973.2 copies/reaction). This bias is likely due to the matrix effect of field samples, which reduces amplification efficiency. As a result, qPCR amplification efficiency for field samples was lower than for standard samples, leading to an underestimation of quantitative results when using standard curves derived from pure gDNA.

Compared to qPCR, ddPCR demonstrate the superior detection performance and quantitative reliability for *P. nicotianae*. The qPCR assays generally requires precise reaction conditions for optimal amplification efficiency, and inadequately optimized qPCR protocols are prone to producing highly variable results (Dijkstra et al., 2014; Taylor et al., 2019).In contrast, digital PCR relies on high-quality primer/probe sets and minimal system optimization, resulting in more robust performance. In our study, the qPCR assay used was well optimized based on the protocol by Li et al (Li et al., 2011), therefore the both methods yielded similar positivity rates for standard samples and root samples with fewer impurities. However, differences were observed in the detection of soil samples with more complex components. The detection sensitivity and quantification accuracy of qPCR were significantly reduced for low-quality test samples. Additionally, ddPCR exhibited a significantly higher positive detection rate than qPCR, particularly in low-concentration soil samples. One of the noteworthy findings was that the negative predictive value (NPV) of ddPCR (47.059%, 95% CI: 33.158–61.400) was higher than that of qPCR (38.889%, 95% CI: 27.841–51.130), indicating a lower rate of false negatives with ddPCR. Since virulence differences among *P. nicotianae* isolates may be associated with variations in pathogen load (GigaScience), ddPCR’s superior sensitivity could provide a more accurate assessment of infection levels, which is critical for studying host-pathogen interactions and disease progression. The different amplification efficiencies of qPCR between standard and field samples resulted in its low sensitivity to detect complex low concentration samples. This difference is also probably responsible for the underestimation of qPCR quantitative results, as evidenced by our results and other reports (Rutledge and Stewart, 2008; Ruijter et al., 2021). Therefore, ddPCR is a more robust quantitative detection method, which is particularly suitable for low-abundance target detection in complex matrices.

Nevertheless, the diagnostic reliability of DNA-based methods (e.g., qPCR and digital PCR) is inherently limited by their inherent inability to discriminate viable pathogens from residual DNA of dead cells, which may lead to overestimate infection levels and distort disease progression evaluations. Subsequent studies would therefore incorporate viability-correlated pathogenicity analysis. In addition, despite digital PCR’s superior sensitivity and absolute quantification capacity, its substantial operational cost (including equipment and consumable costs) currently restricts widespread application (**Supplementary Table S3**). Future research should focus on reducing operational costs, increasing throughput, and improving the practical adoption of digital PCR in routine diagnostics. Furthermore, integrating ddPCR with automated analytical platforms could significantly expand its utility in pathogen surveillance, environmental microbiology, and agricultural disease management.

## Conclusions

This work demonstrates the establishment of a ddPCR method capable of sensitive detection and precise quantification for *P. nicotianae*. While the analytical performance of ddPCR and qPCR is comparable, ddPCR exhibits superior diagnostic performance and higher quantitative accuracy for field samples. Additionally, ddPCR is more sensitive and well-suited for the early detection of complex samples with low pathogen loads. Finally, ddPCR provides a powerful tool for improving disease management strategies, enabling more precise monitoring, timely interventions, and sustainable agricultural practices.

## Data Availability

The original contributions presented in the study are included in the article/**Supplementary Material**. Further inquiries can be directed to the corresponding author/s.

## References

[B1] Genomes and virulence difference between two physiological races of Phytophthora nicotianae. In: GigaScience (Oxford Academic). Available online at: https://academic.oup.com/gigascience/article/5/1/s13742-016-0108-7/2720965 (Accessed 13 Mar 2025). 10.1186/s13742-016-0108-7 PMC4730604 26823972

[B2] Baysal-Gurel F. Bika R. Simmons T. Avin F. (2022). Identification and Management of Phytophthora Aerial Blight Caused by *Phytophthora nicotianae* on *Catharanthus roseus* . Plant Dis. 106, 1271–1277. doi: 10.1094/PDIS-06-21-1342-RE 34854759

[B3] Blaya J. Lacasa C. Lacasa A. Martínez V. Santísima-Trinidad A. B. Pascual J. A.. (2015). Characterization of *Phytophthora nicotianae* isolates in southeast Spain and their detection and quantification through a real-time TaqMan PCR: Characterization of *Phytophthora nicotianae* isolates in southeast Spain. J. Sci. Food Agric. 95, 1243–1251. doi: 10.1002/jsfa.6813 25043929

[B4] Burgess T. I. Edwards J. Drenth A. Massenbauer T. Cunnington J. Mostowfizadeh-Ghalamfarsa R.. (2021). Current status of phytophthora in Australia. Persoonia 47, 151–177. doi: 10.3767/persoonia.2021.47.05 37693794 PMC10486634

[B5] Damgaard M. V. Treebak J. T. (2022). Protocol for qPCR analysis that corrects for cDNA amplification efficiency. STAR Protoc. 3, 101515. doi: 10.1016/j.xpro.2022.101515 35819886 PMC9283931

[B6] Deng L. Zhou J. Sun Y. Hu Y. Qiao J. Liu Z.. (2024). CDKN2A somatic copy number amplification in normal tissues surrounding gastric carcinoma reduces cancer metastasis risk in droplet digital PCR analysis. Gastric Cancer. 27 (5), 986–997. doi: 10.1007/s10120-024-01515-4 38822931

[B7] Dijkstra J. R. van Kempen L. C. Nagtegaal I. D. Bustin S. A. (2014). Critical appraisal of quantitative PCR results in colorectal cancer research: Can we rely on published qPCR results? Mol. Oncol. 8, 813–818. doi: 10.1016/j.molonc.2013.12.016 24423493 PMC5528534

[B8] Dingle T. C. Sedlak R. H. Cook L. Jerome K. R. (2013). Tolerance of droplet-digital PCR vs real-time quantitative PCR to inhibitory substances. Clin. Chem. 59, 1670–1672. doi: 10.1373/clinchem.2013.211045 24003063 PMC4247175

[B9] Dong L. Wang X. Wang S. Du M. Niu C. Yang J.. (2020). Interlaboratory assessment of droplet digital PCR for quantification of *BRAF* V600E mutation using a novel DNA reference material. Talanta 207, 120293. doi: 10.1016/j.talanta.2019.120293 31594564

[B10] Hou Y. Chen S. Zheng Y. Zheng X. Lin J.-M. (2023). Droplet-based digital PCR (ddPCR) and its applications. TrAC Trends Analytical Chem. 158, 116897. doi: 10.1016/j.trac.2022.116897

[B11] Hu B. Tao Y. Shao Z. Zheng Y. Zhang R. Yang X.. (2021). A comparison of blood pathogen detection among droplet digital PCR, metagenomic next-generation sequencing, and blood culture in critically ill patients with suspected bloodstream infections. Front. Microbiol. 12. doi: 10.3389/fmicb.2021.641202 PMC8165239 34079528

[B12] Kalantar K. L. Carvalho T. de Bourcy C. F. A. Dimitrov B. Dingle G. Egger R.. (2020). IDseq—An open source cloud-based pipeline and analysis service for metagenomic pathogen detection and monitoring. GigaScience 9, giaa111. doi: 10.1093/gigascience/giaa111 33057676 PMC7566497

[B13] Kralik P. Ricchi M. (2017). A basic guide to real time PCR in microbial diagnostics: definitions, parameters, and everything. Front. Microbiol. 8. doi: 10.3389/fmicb.2017.00108 PMC5288344 28210243

[B14] Kroll M. (2004). Protocols for determination of limits of detection and limits of quantitation: approved guideline.

[B15] Li M. Asano T. Suga H. Kageyama K. (2011). A Multiplex PCR for the Detection of *Phytophthora nicotianae* and P. cactorum, and a Survey of Their Occurrence in Strawberry Production Areas of Japan. Plant Dis. 95, 1270–1278. doi: 10.1094/PDIS-01-11-0076 30731689

[B16] Li M. Inada M. Watanabe H. Suga H. Kageyama K. (2013). Simultaneous Detection and Quantification of *Phytophthora nicotianae* and P. cactorum, and Distribution Analyses in Strawberry Greenhouses by Duplex Real-time PCR. Microb. Environ. 28, 195–203. doi: 10.1264/jsme2.ME12177 PMC4070668 23614901

[B17] Ma X. Xu J. Zhao X. Qu L. Gao Y. Huang W.. (2024). Selenium improves the control efficacy of phytophthora nicotianae by damaging the cell membrane system and promoting plant energy metabolism. J. Agric. Food Chem. 72 (9), 5073–5087. doi: 10.1021/acs.jafc.3c07277 38377432

[B18] Maheshwari Y. Selvaraj V. Hajeri S. Yokomi R. (2017). Application of droplet digital PCR for quantitative detection of Spiroplasma citri in comparison with real time PCR. PloS One 12, e0184751. doi: 10.1371/journal.pone.0184751 28910375 PMC5599046

[B19] Mellert H. S. Jackson L. Tompkins C. Lodge A. Pestano G. A. (2017). Development of DDPCR blood-based diagnostic tests that simultaneously measure mRNA expression from immune and cancer cells. J. Clin. Oncol. 35, 22–22. doi: 10.1200/JCO.2017.35.7_suppl.22

[B20] Meng J. Ji H. Chen L. Liu A. (2022). Comparison of droplet digital PCR and metagenomic next-generation sequencing methods for the detection of human herpesvirus 6B infection using cell-free DNA from patients receiving CAR-T and hematopoietic stem cell transplantation. Infect. Drug Resist. 15, 5353–5364. doi: 10.2147/IDR.S379439 36110128 PMC9469937

[B21] Pandey R. (2023). Controlling tobacco diseases: an overview of black shank and fusarium wilt. Int. J. Appl. Sci. Biotechnol. 11, 1–7. doi: 10.3126/ijasbt.v11i1.52440

[B22] Piombo E. Abdelfattah A. Droby S. Wisniewski M. Spadaro D. Schena L. (2021). Metagenomics approaches for the detection and surveillance of emerging and recurrent plant pathogens. Microorganisms 9, 188. doi: 10.3390/microorganisms9010188 33467169 PMC7830299

[B23] Raftoyannis Y. Dick M. W. (2006). Zoospore encystment and pathogenicity of Phytophthora and Pythium species on plant roots. Microbiological Res. 161, 1–8. doi: 10.1016/j.micres.2005.04.003 16338584

[B24] Riudavets M. Lamberts V. Auclin E. Aldea M. Vasseur D. Jovelet C.. (2021). 22P Clinical utility of ddPCR for detection of sensitizing and resistance EGFRm in pts with advanced NSCLC. J. Thorac. Oncol. 16, S707–S708. doi: 10.1016/S1556-0864(21)01864-5

[B25] Roman-Reyna V. Crandall S. G. (2024). Seeing in the dark: a metagenomic approach can illuminate the drivers of plant disease. Front. Plant Sci. 15. doi: 10.3389/fpls.2024.1405042 PMC11269093 39055364

[B26] Rotondo J. C. (2020). Simultaneous detection and viral DNA load quantification of different human papillomavirus types in clinical specimens by the high analytical droplet digital PCR method. Front. Microbiol. 11. doi: 10.3389/fmicb.2020.591452 PMC7710522 33329471

[B27] Ruijter J. M. Barnewall R. J. Marsh I. B. Szentirmay A. N. Quinn J. C. van Houdt R.. (2021). Efficiency correction is required for accurate quantitative PCR analysis and reporting. Clin. Chem. 67, 829–842. doi: 10.1093/clinchem/hvab052 33890632

[B28] Rutledge R. G. Stewart D. (2008). Critical evaluation of methods used to determine amplification efficiency refutes the exponential character of real-time PCR. BMC Mol. Biol. 9, 96. doi: 10.1186/1471-2199-9-96 18973660 PMC2587475

[B29] Sidstedt M. Rådström P. Hedman J. (2020). PCR inhibition in qPCR, dPCR and MPS—mechanisms and solutions. Anal. Bioanal Chem. 412, 2009–2023. doi: 10.1007/s00216-020-02490-2 32052066 PMC7072044

[B30] Taylor S. C. Nadeau K. Abbasi M. Lachance C. Nguyen M. Fenrich J. (2019). The ultimate qPCR experiment: producing publication quality, reproducible data the first time. Trends Biotechnol. 37, 761–774. doi: 10.1016/j.tibtech.2018.12.002 30654913

[B31] Tholen D. W. Linnet K. Kondratovich M. Armbruster D. A. Garrett P. E. Jones R. L.. (2004). Protocols for determination of limits of detection and limits of quantitation; approved guidelines. EP17-A. 24, No.34.

[B32] Vanegas-Villa D. M. Navarro-Álzate R. A. Afanador-Kafuri L. Gutiérrez-Monsalve J. A. Morales-Osorio J. G. Uribe-Soto S. I.. (2020). Effect of interaction between Phytophthora nicotianae and Meloidogyne spp. on the productivity and quality of tobacco plants (Nicotiana tabacum. Nematology 22, 1179–1192. doi: 10.1163/15685411-bja10021

[B33] Wang D. Jiao X. Jia H. Cheng S. Jin X. Wang Y.. (2022). Detection and quantification of Verticillium dahliae and V. longisporum by droplet digital PCR versus quantitative real-time PCR. Front. Cell Infect. Microbiol. 12. doi: 10.3389/fcimb.2022.995705 PMC9441566 36072220

[B34] Wang D. Wang S. Du X. He Q. Liu Y. Wang Z.. (2022). ddPCR surpasses classical qPCR technology in quantitating bacteria and fungi in the environment. Molecular Ecology Resources. 22, 2587–2598. doi: 10.1111/1755-0998.13644 35587727

[B35] Zafeiriadou A. (2024). Evaluation of PCR-enhancing approaches to reduce inhibition in wastewater samples and enhance viral load measurements. Sci. Total Environ. 955, 176768. doi: 10.1016/j.scitotenv.2024.176768 39393702

[B36] Zhang S. Liu S. Zhang J. Reiter R. J. Wang Y. Qiu D.. (2018). Synergistic anti-oomycete effect of melatonin with a biofungicide against oomycetic black shank disease. J. Pineal Res. 65, e12492. doi: 10.1111/jpi.12492 29575191

[B37] Zhao F. Maren N. A. Kosentka P. Z. Liao Y.-Y. Lu H. Duduit J. R.. (2021). An optimized protocol for stepwise optimization of real-time RT-PCR analysis. Hortic. Res. 8, 1–21. doi: 10.1038/s41438-021-00616-w 34333545 PMC8325682

[B38] Zhu K. Suttner B. Pickering A. Konstantinidis K. T. Brown J. (2020). A novel droplet digital PCR human mtDNA assay for fecal source tracking. Water Res. 183, 116085. doi: 10.1016/j.watres.2020.116085 32750535 PMC7495096

