# Supplementary material for: Development and validation of a droplet digital PCR assay for sensitive detection and quantification of Phytophthora nicotianae
Source: Front Plant Sci. 2025 Apr 25;16:1573949. doi: 10.3389/fpls.2025.1573949 (PMC12061998; doi:10.3389/fpls.2025.1573949)
Supplement: Supplementary file 1 [file DataSheet1.docx]

**Supplementary Information**

Table S1. Specificity test for the ddPCR assay.

| **Name** | **Ct value** |
| --- | --- |
| *P. capsici* | ND |
| *P. cactorum* | ND |
| *P. ramorum* | ND |
| *P. infestans* | ND |
| *P. citrophthora* | ND |
| *P. cryptogea* A1 | ND |
| *P. cryptogea* A2 | ND |
| *P. cinnamomi* | ND |
| *Pythium* spp. | ND |
| *P. ultimum* | ND |
| *P. aphanidermatum* | ND |
| *Pythium myriotylum* | ND |
| *Verticillium albo-atrum* | ND |
| *Verticillium dahliae* | ND |
| *Pythium helicoid* | ND |

ND: not detected.

**Table S2.** Upper 15 Blank Values (copies/reaction) for determining the LoB.

| Ranks | Blank values | |
| --- | --- | --- |
|  | qPCR | ddPCR |
| 60 | 7.17 | 6.2 |
| 59 | 4.15 | 3.4 |
| 58 | 3.96 | 3.2 |
| 57 | 2.14 | 3 |
| 56 | 1.28 | 2.6 |
| 55 | 1.27 | 2.4 |
| 54 | 1.27 | 2.4 |
| 53 | 0.84 | 1.4 |
| 52 | 0.83 | 0 |
| 51 | 0.37 | 0 |
| 50 | 0.11 | 0 |
| 49 | 0.09 | 0 |
| 48 | 0 | 0 |
| 47 | 0 | 0 |
| 46 | 0 | 0 |
| 45 | 0 | 0 |





**Figure S1**. LoB of qPCR and ddPCR. Recorded distributions of 60 blank measurements for determination of LoB of the *P. nicotianae* copy number per reaction.

**Table S3.** Cost and Time Comparison for Metagenomics, ddPCR, and qPCR

| Method | Equipment Cost ($) | Reagent Cost per Sample ($) | Time Required (per run) |
| --- | --- | --- | --- |
| Metagenomics | Very High  (~200,000–500,000) | High  (~100–300) | Days to weeks |
| Digital PCR | High  (~100,000-300,000) | Moderate  (~5–15)0 | ~2.5–3 hours |
| qPCR | Moderate  (~20,000–50,000) | Low  (~1–5) | ~2–3 hours |
